# Nucleic Acid Sandwich Hybridization Assay with Quantum Dot-Induced Fluorescence Resonance Energy Transfer for Pathogen Detection

**DOI:** 10.3390/s121216660

**Published:** 2012-12-04

**Authors:** Cheng-Chung Chou, Yi-Han Huang

**Affiliations:** Department of Life Science and Center for Nano Bio-Detection, National Chung-Cheng University, Chia-Yi 62102, Taiwan; E-Mail: je091410show@hotmail.com

**Keywords:** Alexa Fluor 660, avian influenza virus H5N1, fluorescence resonance energy transfer (FRET), indium tin oxide (ITO), quantum dot 655 (QD655), sandwich hybridization

## Abstract

This paper reports a nucleic acid sandwich hybridization assay with a quantum dot (QD)-induced fluorescence resonance energy transfer (FRET) reporter system. Two label-free hemagglutinin H5 sequences (60-mer DNA and 630-nt cDNA fragment) of avian influenza viruses were used as the targets in this work. Two oligonucleotides (16 mers and 18 mers) that specifically recognize two separate but neighboring regions of the H5 sequences were served as the capturing and reporter probes, respectively. The capturing probe was conjugated to QD655 (donor) in a molar ratio of 10:1 (probe-to-QD), and the reporter probe was labeled with Alexa Fluor 660 dye (acceptor) during synthesis. The sandwich hybridization assay was done in a 20 μL transparent, adhesive frame-confined microchamber on a disposable, temperature-adjustable indium tin oxide (ITO) glass slide. The FRET signal in response to the sandwich hybridization was monitored by a homemade optical sensor comprising a single 400 nm UV light-emitting diode (LED), optical fibers, and a miniature 16-bit spectrophotometer. The target with a concentration ranging from 0.5 nM to 1 μM was successfully correlated with both QD emission decrease at 653 nm and dye emission increase at 690 nm. To sum up, this work is beneficial for developing a portable QD-based nucleic acid sensor for on-site pathogen detection.

## 1. Introduction

Zoonoses have continuously threatened public health and economics worldwide. A notable example is avian influenza (AI), caused by the infection of type A influenza viruses of the family Orthomyxoviridae. Avian influenza is a severe disease in domestic poultry, and its outbreak often results in high mortality in flock and leads to substantial economic losses [1]. Since the outbreak of the highly pathogenic H5N1 avian influenza in Hong Kong in 1997, growing evidence has been showing that avian influenza viruses (AIVs) can cross the species barrier to directly infect humans [2,3]. According to the World Organization for Animal Health, H5N1 AIVs have induced over hundred human deaths in Asia in the past few years and created a worldwide public health problem. Early identification of the highly pathogenic AIVs and other zoonotic pathogens in the field can help control zoonosis spread and reduce the risk of development into an epidemic. Therefore, rapid, highly specific and sensitive detection methods for routine surveillance are keys to the efficient prevention and control of AIVs.

Conventional diagnosis of influenza viruses is based on viral culture and ELISA assays. The former is quite sensitive, but time-consuming and labor-intensive; instead, the latter is rapid, but with limited specificity [4]. The employment of reverse transcriptase-PCR (RT-PCR) techniques has enabled the rapid and accurate diagnosis of AIV infection through detection of specific viral genomic nucleic acid sequences [5]. However, the assay requires multiple sample manipulations, thus increasing the risk of carryover contamination and lowering the chance of the implementation for routine testing programs. Recently, more advanced real-time RT-PCR assays have been used for AIV detection [6], which enables amplification of nucleic acids and detection of the amplified products through specific probes at the same. Compared to RT-PCR, this method displays better sensitivity and specificity, reduces the potential for sample cross contamination, and enables quantitative identification. However, high equipment cost and specific technical training requirements limit the usefulness and availability of this assay as routine AIV surveillance. Microarray technology has also been applied to the detection of influenza A viruses [7,8] but generally lacks the necessary sensitivity and rapidity for point-of-care diagnosis. AIVs, unlike other infectious viruses, require on-site surveillance to implement early and efficient control strategies for preventing further spread or new outbreak [9]. None of the aforementioned detection tools are fully qualified for the requirement, which has highlighted the necessity to improve existing tests or to develop new molecular technologies for on-site AIV detection. To achieve this goal, we propose a prototype of portable detection platform that is not only well-suited for on-site nucleic acid diagnosis but also compatible with DNA or RNA analyses in a laboratory. The platform is on the basis of a “nucleic acid sandwich hybridization” assay with a reporter detection system by quantum dot (QD)-induced fluorescence resonance energy transfer (FRET) that is monitored on a disposable, temperature-adjustable indium tin oxide (ITO) glass slide.

Compared to single-probe recognition, sandwich hybridization assays using two distinct oligonucleotide probes to recognize a nucleic acid sequence show remarkable target specificity and eliminate the need of target labeling. As a result, sandwich hybridization has been applied to nucleic acid detection for years [10,11]. Moreover, sandwich hybridization is compatible with FRET detection [12] by anchoring a donor fluorophore and an acceptor fluorophore on the two oligonucleotide probes, respectively. FRET, an emission-shift phenomenon, is a non-contact, energy transfer between two fluorophores, occurring only when a donor fluorophore and an acceptor fluorophore have both spatial and spectral proximity. Hence, FRET can introduce secondary specificity for target-probe recognition and has therefore been employed as a molecular beacon tool for *in situ* monitoring a variety of biomolecular binding events such as protein detection and nucleic acid hybridization [12]. A number of rapid and sensitive proximity-based biosensors have been developed accordingly [13–15]. Recently, more and more evidences show that FRET detection can be furthered improved using QDs, colloidal semiconductor nanoparticles resembling donor fluorophores, since QDs can be efficiently excited by light sources (e.g., UV region) far away from the excitation spectrums of FRET acceptors to avoid direct excitation of the acceptor [16,17]. Moreover, the narrow and symmetric emission spectra of QDs result in highly FRET donor-acceptor spectral overlap to reduce crosstalk between donor and acceptor channels [18]. Currently, the QD-FRET sensing system associated with sandwich hybridization has been widely used for the selective detection of nucleic acids. For instance, the use of immobilized QDs as FRET donors has been successfully applied for solid-phase detection of nucleic acid hybridization [19,20]. Additionally, using a single color of QD to induce FRET associated with two-color coincidence detection has been developed for multiplex diagnosis of HIV-1 and HIV-2 at single-molecule level in the solution [21], in which specialized instrumentation and methodology (e.g., confocal fluorescence spectroscopy) will be required to meet the challenges of detecting single molecules. Alternatively, QDs have been served as FRET acceptors, in which chemiluminescence, generated by the hemin/G-quadruplex, acts as a FRET donor for inducing chemiluminescence energy transfer (CRET) to the QDs. The use of QDs functionalized with different nucleic acids allows for multiplexed CRET-based detection of different DNA targets [22]. Considering the above facts, we have designed and investigated a sandwich hybridization assay with a QD-induced FRET reporter system in homogeneous solution free of sample isolation or probe immobilization for nucleic acid detection.

**Figure 1.**
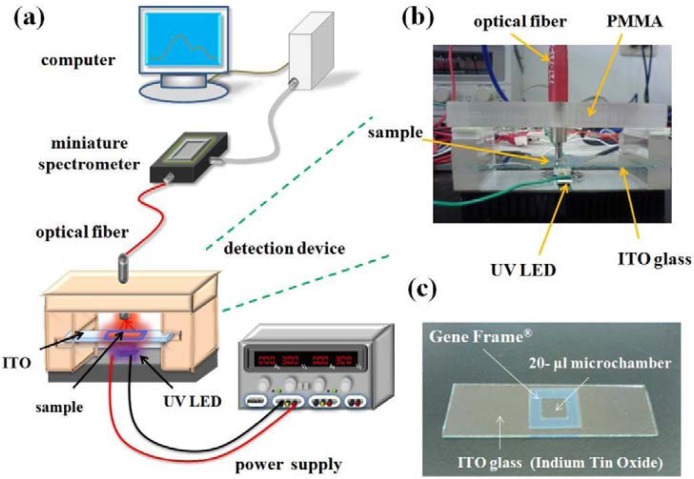
The homemade FRET detection system based on a single 400 nm UV LED, a disposable ITO glass slide, a miniature 16-bit CCD-array spectrophotometer, and optical fibers: (**a**) schematic diagram of the detection system setup; (**b**) picture of the detection device setup (the main body of the device was made of PMMA with dimensions of 8.4 × 4.5 × 4.3 cm^3^); (**c**) ITO with a transparent, 20 μL microchamber created by the Microarray Gene Frame^®^.

In addition to the assay format, the sensing hardware is another crucial factor for successful on-site nucleic acid sensor development. To date, quite some researchers have reported portable biosensors for the “field testing” of nucleic acid targets [23–25]. These works imply that a good on-site nucleic biosensor should be user-friendly, low-weight, battery-powered, robust and free of contaminations. The last criterion is particularly important since nucleic acid contaminants are ubiquitous. Accordingly, we assembled an optical sensor with an UV LED, optical fibers and a miniature CCD spectrophotometer for the QD-induced FRET detection (Figure 1(a,b)), and the sample solution is placed and monitored on a disposable, temperature-adjustable ITO glass slide (Figure 1(c)). Having high conductivity and optical transparency, ITO was recently reported to be an ideal electro-heating element for the development of a static-chip, portable real-time PCR system [26]. It was shown that precise temperature control, rapid temperature ramping and *in situ* fluorescence monitoring could all be achieved with an ITO glass substrate. As a result, ITO was used as the detection interface in the present work to function like a test strip for monitoring the QD-induced FRET signal derived from the sandwich hybridization between the probes and the target.

To demonstrate the detection platform proposed above, a label-free, 60-mer conserved hemagglutinin H5 sequence derived from a literature study [27] was initially used as the target for assay optimization. Two specially designed oligonucleotide probes, conjugated to QD655 (FRET donor) and labeled with Alexa Fluor 660 (FRET acceptor) respectively, were used for the sandwich hybridization with the target sequence. The objective of this work is to correlate the target concentration with both QD emission decrease and dye emission increase. To this end, the factors that affect the QD-induced FRET performance in the sandwich hybridization were investigated. The assay condition was optimized as well to attain high correlation between the FRET signal and the target concentration. Finally, a partial H5 cDNA fragment (630-nt) complementary to the above FRET probe pair was spiked into a sample buffer with a huge background of human nucleic acids to demonstrate the clinical feasibility of the sandwich hybridization assay.

## 2. Experimental Section

### 2.1. Target Sequences and Oligonucleotide Probes

A label-free, 60-mer conserved H5 sequence (5′-AAG ATA GAC CAG CTA CCA TGA TTG CCA GTG CTA GGG AAC TTG CCA CTG TTG AAT AAA TTG-3′) was used to demonstrate the feasibility of the sandwich hybridization assay. Two oligonucleotides that specifically recognize two separate but neighbor regions of the target sequence were served as the capturing probe (5′-CAG TG G CGA G(T/C)T CCC T-3′) and the reporter probe (5′-ACT GGC AAT CAT GGT (A/G)GC-3′), respectively. The 3′ end of the capturing probe was modified with a thiol (-SH) linker for conjugation with Qdot 655 ITK™ amino (PEG) quantum dots, namely QD655 (Invitrogen, Carlsbad, CA, USA) (FRET donor). The 5′ end of the reporter probe was labeled with an Alexa Fluor 660 dye (FRET acceptor). A 60-mer hemagglutinin H1 sequence (5′-AGT ACC AAT GAA CTG GCG ACA GTT GAA TAG ATC GCC AAA ATC TGG TAA ATC CTT GTT GAT-3′) with 40.6% sequence similarity to the 60-mer H5 target was used as a non-target control in this study. All the sequences with and without terminal modifications were all synthesized and purified by Integrated DNA Technology Inc. (Coraville, IA, USA). The construction of a 630-nt H5 cDNA fragment is described as follows. First, the full-length H5 gene sequence from HK/156/97/H5N1 AIV (accession number AF028709) was synthesized by overlapping oligonucleotide assembly approach, in which 88 oligonucleotides (40-mer) encoding both strands of the H5 sequence were combined and assembled in a two-stage PCR protocol [28]. The H5 gene was then *in vitro* transcribed into its corresponding RNA, from which a 630-nt cDNA fragment, ranging from nt 1,112 to 1,741 in the H5 gene, was synthesized by reverse transcription. For spiking experiments, total nucleic acids from human bronchial epithelial cells (BEAS-2B cells) were extracted by High Pure Viral Nucleic Acid Isolation kit (Roche Diagnostics, Mannheim, Germany).

### 2.2. Conjugation of Capturing Probes with Quantum Dots

The protocol used for conjugation of capturing probes with Qdot 655 ITK™ amino (PEG) quantum dots (QD655) was modified from the steps reported in the literature [29] and is described briefly as follows. At first, 62.5 μL of QD655 (8 μM) was buffer exchanged to 250 μL of Buffer A (50 mM sodium phosphate and 150 mM sodium chloride, pH 7.2) using Amicon Ultra-4 (100 kDa cutoff) centrifugal filter units (4,000×g at 4 °C for 15 min). Then 250 μL of sulfo-SMCC crosslinker (2 mM) (Pierce, Rockford, IL, USA, in 1,000-fold excess, in Buffer A) was added into the QD suspension and allowed to react at room temperature for 1 h. The resulting sulfo-SMCC crosslinked QDs were filtered by a NAP-5 column (GE Healthcare, Buckinghamshire, UK) to remove excess cross-linker and eluted with 1 mL of Buffer B (50 mM sodium phosphate, 150 mM sodium chloride, 10 mM EDTA, pH 7.2). 5 μL of 1 mM capturing probes with 3′ thiol linkers were mixed with 245 μL of 0.1 M dithiothreitol at room temperature for 1 h. The mixture was then filtered by a NAP-5 column into 0.7 mL of Buffer A and reacted with the above-mentioned sulfo-SMCC crosslinked QDs at 4 °C overnight. In theory, the capturing probes were conjugated to QD655 in a molar ratio of 10:1 (probe:QD).

**Figure 2.**
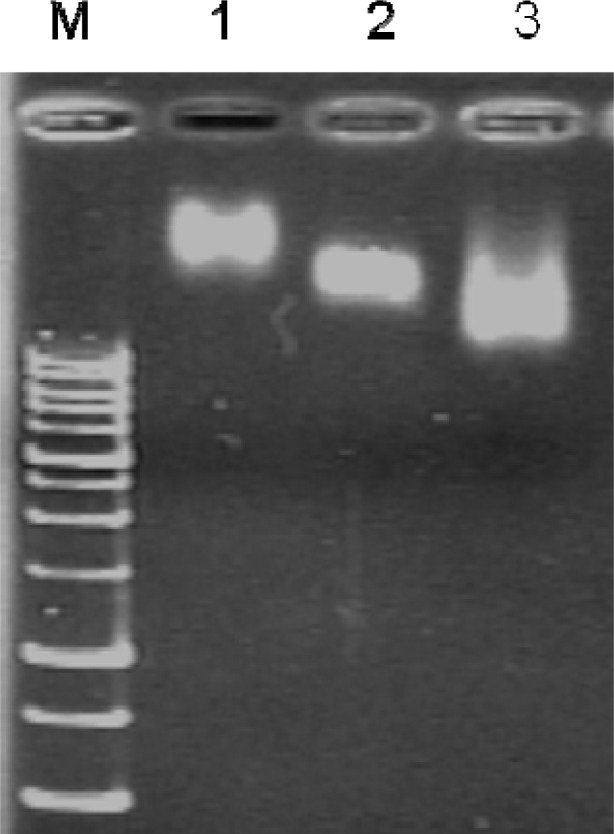
Mobility shift assay of QD655 conjugation with capturing probes. **M**: 1 kb DNA ladder, **lane 1**: QD655 particles only, **lane 2**: QD655 modified with sulfo-SMCC crosslinker, **lane 3**: sulfo-SMCC modified QD655 conjugated with capturing probes. The QD655 particles surrounded with positively charged amine-derivatized PEG do not migrate very quickly (**lane 1**). The binding of sulfo-SMCC on the QD655 increases the mobility of the conjugated particles since sulfo-SMCC possesses a negatively charged sulfonate group on its NHS ring (**lane 2**). The binding of capturing DNA probes to sulfo-SMCC modified QD655 particles further increases the mobility of the conjugates due to the additional negative charge introduced by the oligonucleotide probes (**lane 3**), verifying the conjugation of QD655 with capturing probes.

The conjugated products were washed twice with 4 mL of phosphate buffered saline (PBS), twice with a 4 mL of high salt buffer (1.0 M sodium chloride, 100 mM sodium citrate, pH 7.2), and then twice again with 4 mL of PBS using Amicon Ultra-4 (100 kDa cutoff) centrifugal filter units according to the vender’s recommendation. The success of QD655 conjugation was confirmed by 2% agarose gel electrophoresis (Figure 2). The QD conjugates were then blocked by 0.05% denatured bovine serum albumin at 60 °C for 1 h [30,31] to prevent false positive FRET signals due to possible non-specific adsorption of fluorophore-labeled oligonucleotides on the QDs. Finally, the blocked QD-conjugated capturing probes were purified using Amicon Ultra-4 100 kDa cutoff concentrator and resuspended in 50 μL of PBS.

### 2.3. Nucleic Acid Sandwich Hybridization

20 μL mixtures of the capturing probes (conjugated with QD665), the reporter probes (labeled with Alexa Fluor 660 dye) and the H5 target DNA at different molar ratios were prepared in a sample buffer (20 mM Tris-HCl, pH 8.4, 50 mM KCl, 7 mM MgCl_2_) and transferred onto an ITO glass slide for 1.5 h of incubation at 37 °C before FRET detection. The concentrations of capturing and reporter probes used were quantified by the concentrations of the conjugated QD (denoted as [QD]) and the labeled Alexa Fluor 660 dye (denoted as [dye]), respectively (note: in the ideal case, the capturing probe-to-QD ratio and the reporter probe-to-dye ratio are 10:1 and 1:1, respectively). The concentration of H5 target is denoted as [H5]. The concentrations of QD, Alexa Fluor dye and target DNA were all quantified with a UV-visible spectrophotometer (WPA S2100, Biochrom Ltd., Cambridge, UK).

### 2.4. Detection of Sandwich Hybridization with QD-Induced FRET

The QD-induced FRET spectra of the capturing probe/H5 target/reporter probe complex were monitored by a homemade, low weight and cost-effective optical sensor system. The optical sensor system was constructed based on a single UV LED featuring a narrow emission band between 395 and 410 nm (EFEV-1AE1, flux(lm) = 160 mW, V_f_ = 3.0–4.0 V, Edison Opto, Taipei, Taiwan), an ITO glass slide having dimensions 7.62 × 2.54 × 0.1 cm^3^ with transmittance ≥85% in 400–700 nm and R_sh_ = 7 ohm/sq (Ritdisplay Corporation, Hsin-Chu, Taiwan), and a miniature 16-bit CCD-array spectrophotometer (USB 4000, Ocean Optics, Dunedin, FL, USA). An optical fiber with a diameter of 0.6 mm (QP600-2-UV-VIS multimode fiber, Ocean Optics) was striped and trimmed and then connected into a standard SMA 905 connector (Ocean Optics) to transmit the light. Moreover, additional optical filters (Newport Corp., Irvine, CA, USA) on excitation (500 nm shortpass) and emission (500 nm longpass) sites were used to reduce the background signal. Figure 1 shows the setup of the FRET detection device. The 20 μL, transparent microchamber on the ITO glass slide was constructed by applying a sticky 25 μL Microarray Gene Frame^®^ (AB-0576, area = 1 × 1 cm^2^, thickness = 0.25 mm, ABgene, Epsom, UK) onto the ITO conductive surface along with a transparent, plastic cover slip. The assembly of Gene Frame^®^ and cover slip were thermally stable and remained well-sealed up to 97 °C. The hybridization mixture was transferred to the microchamber with a micropipette. Then the miniature CCD-array spectrophotometer, connected to a laptop computer, was used to collect the emission spectra detected by the optical sensor system.

## 3. Results and Discussion

### 3.1. Sandwich Hybridization Assay with a QD-Induced FRET Reporter System

**Figure 3.**
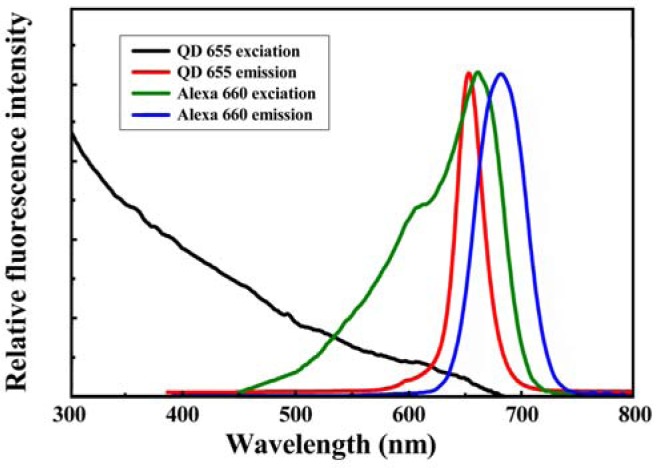
Comparison between the excitation and emission spectra of QD655 and Alexa Fluor 660. The emission maxima of QD655 and Alexa Fluor 660 occur at 653 and 690 nm, respectively. The excitation spectra were measured by the lab-grade spectrophotometer with quartz cuvettes; the emission spectra were collected by the homemade UV LED/ITO/CCD spectrometer optical sensor.

**Figure 4.**
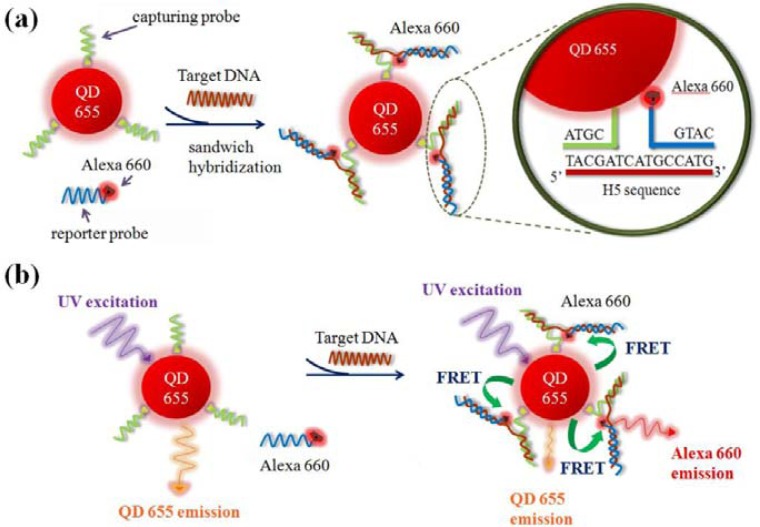
Schematic illustration of the sandwich hybridization assay with a QD-induced FRET reporter system for H5 target DNA detection: (**a**) sandwich hybridization with label-free H5 sequence (target) by the capturing probes conjugated on QD655 (FRET donor) and the reporter probes labeled with Alexa Fluor 660 (FRET acceptor); (**b**) FRET emission shift before and after the sandwich hybridization.

In general, the application of FRET technique to quantification of a binding event can be carried out by either the emission-shift method or the donor-quenching method [32]. The emission-shift strategy can exhibit two-dimensional signals (decrease in QD emission and increase in dye emission) and was used in this work. Because the emission maximum of QD655 and the excitation maximum of Alexa Fluor 660 occur at 653 and 660 nm, respectively (Figure 3), the high overlap between the spectra of QD655 (donor) emission and Alexa Fluor 660 (acceptor) excitation could induce high-efficiency FRET, so the QD-dye FRET pair was used as the reporter system in the sandwich hybridization assay. Figure 4 illustrates the molecular design for the sandwich hybridization assay. As shown in Figure 4(a), capturing probes are conjugated to QD655, while each reporter probe carries an Alexa Fluor 660 dye label. If the sandwich hybridization occurs, the binding event between the two probes and the target will cause spatial approaching between QD and dye and then induce a FRET effect under UV excitation (Figure 4(b)). Thus, the FRET reporter system can turn target recognition into a fluorescence shift signal and the extent of the FRET emission shift should depend on the target concentration—An increase in target concentration leads to a decrease in QD655 emission but an increase in Alexa Fluor 660 emission. Here the FERT efficiency (E, %) and FRET value (R), as defined below, are calculated to quantify the percentage of energy transfer and the extent of the FRET emission shift, respectively. The optimal assay condition for H5 detection is determined accordingly:

(1) $$\text{E}\left(\%\right)=\left(1-\frac{\text{emission}\;\text{of}\;\text{QD}\;\text{when}\;\text{FRET}\;\text{occurs}}{\text{emission}\;\text{of}\;\text{QD}\;\text{without}\;\text{FRET}}\right)\times 100\%$$

(2) $$\text{R}=\frac{\text{emission}\;\text{of}\;\text{Alexa}\;\text{Fluor}\;660\;\text{at}\;690\;\text{nm}}{\text{emission}\;\text{of}\;\text{QD}655\;\text{at}\;653}$$

### 3.2. Toward the Target Detection with the FRET Emission Shift

**Figure 5.**
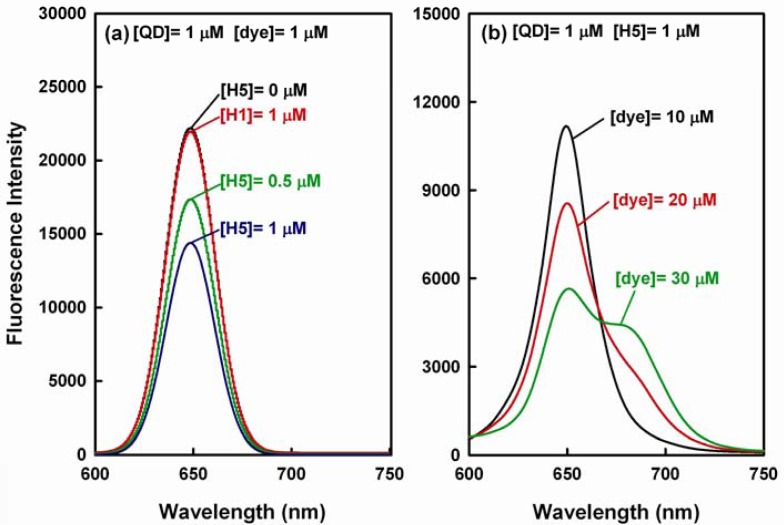
The emission spectra of the sandwich hybridization with a high FRET donor concentration ([QD] = 1 μM): (**a**) hybridization with different amounts of H5 target ([H5] = 0, 0.5, and 1 μM) and a fixed amount of FRET acceptor ([dye] = 1 μM) and a negative control study in which non-target 60-mer H1 sequence ([H1] = 1 μM) was hybridized with QD-dye FRET pair ([QD] = [dye] = 1 μM) to evaluate the detection specificity of the QD-dye FRET pair; (**b**) hybridization with a fixed amount of H5 target ([H5] = 1 μM) and different amounts of FRET acceptor ([dye] = 10, 20, and 30 μM).

In theory, the aforementioned sandwich hybridization should result in a FRET emission shift from 653 nm to 690 nm, but we found that a reasonable high FRET value as well as a detectable emission shift occurs when the sandwich hybridization is done with a high dye-to-QD ratio. At the beginning of our study, we employed a high concentration of QD655 donor ([QD] = 1 μM) and Alexa Fluor 660 dye ([dye] = 1 μM) in the sandwich hybridization assay. As shown in Figure 5(a), the QD emission obviously decreases with the increase in target concentration, but the QD emission decrease does not turn into an observable dye emission increase (for [H5] = 1 μM, E = 34.8% and R = 0.05; the background FRET value is about 0.02). To investigate whether the QD emission decrease is due to the hybridization of QD-dye FRET pair with the H5 target, we carried out a negative control study in which 60-mer non-target H1 sequence ([H1] = 1 μM) was hybridized with QD-dye probe pair ([QD] = [dye] = 1 μM). The emission spectrum in the hybridization is not different from that for QD-dye pair ([QD] = [dye] = 1 μM) without the addition of H5 target ([H5] = 0 μM). This result indicates that the QD-dye FRET probe pair shows no significant response to the H1 sequence and the aforementioned decrease in QD emission should be due to the hybridization of the FRET pair to the H5 target.

Figure 5(b) displays the spectra with 10–30 μM dye and 1 μM QD for 1 μM target and shows that an obvious emission shift is detected when the dye concentration is 30 μM (In this case, E = 74.9% and R = 0.76). Thereafter, the FRET effect gradually approached to saturation since the further increase in dye concentration >30 μM did not significantly enhance the FRET emission shift signal (data not shown). The aforementioned result indicates that a high dye-to-QD ratio can enhance both the FRET efficiency and FRET value. Thus, if both the QD emission decrease at 653 nm and the dye emission increase at 690 nm are to be correlated to the target concentration in the sandwich hybridization assay, a high dye-to-QD ratio is required. Presumably, the corresponding reason is twofold. First, in theory, a high dye-to-QD ratio causes more acceptor fluorophores gathering around a QD donor particle during sandwich hybridization and enhances the FRET efficiency and thus the FRET value. Second, a reporter probe molecule carries only one Alexa Fluor dye. That is, the dye concentration is equal to the concentration of the reporter probe. On the other hand, the capturing probes were conjugated to QD655 in a molar ratio of 10:1, so the capturing probe concentration is considered 10 times of the QD concentration in the ideal case. The actual active sites on each QD surface that allow anchoring DNA probes might be even higher than 10 [33]. Hence, a high dye-to-QD ratio is considered favored, because it gives a comparable or excess quantity of reporter probes for the capturing probes on QDs. Thus, a dye-to-QD ratio of 30:1 was used in the subsequent sandwich hybridizations in order to obtain FRET emission shift spectra information.

### 3.3. Correlation of Target Concentration with FRET Emission Shift Data

**Figure 6.**
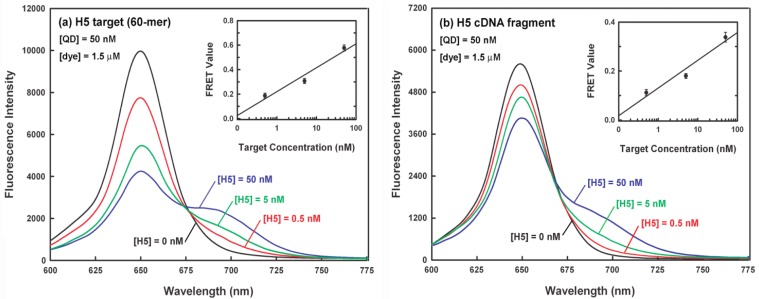
FRET detection of different amounts of (**a**) 60-mer H5 target (0–50 nM) and (**b**) 630-nt H5 cDNA fragment target (0–50 nM) with 50 nM FRET donor (QD) and 1.5 μM FRET acceptor (dye). The insets of (**a**) and (**b**) display the dose response relationship between the average FRET value from triplicate experiments and the target concentration.

Figure 6(a) shows a representative spectral dataset for a series of sandwich hybridization assays with 50 nM QD, 1.5 μM dye (*i.e.*, a dye-to-QD ratio of 30:1) and different concentrations of 60-mer H5 target (0–50 nM). It can be seen that the target DNA concentration can be correlated with both signal decrease in QD emission at 653 nm and signal increase in dye emission at 690 nm. The average FRET values from triplicate experiments for the target concentrations at 0.5 nM, 5 nM and 50 nM are 0.21, 0.35, and 0.59, respectively and their corresponding relative standard deviations (RSD) are 11.6, 7.1, and 4.9%, respectively (inset of Figure 6(a)). The correlation coefficient between the target concentration and FRET value is 0.975. Since the background FRET value (when the target is absent) is 0.08 (RSD = 13.4%), the estimated limit of detection (LOD), a concentration corresponding to a FRET value three standard deviations above the background FRET value, is about 0.27 nM for 50 nM of QD donor used in the sandwich hybridization, which is comparable to or better than several QD-based FRET bioassays with LODs of 1–12 nM [19,20,31,34].

### 3.4. Demonstration of Clinical Feasibility for the QD-Induced FRET Reporter System

Accurate detection of target in complex mixture is highly required in clinical diagnosis. The nucleic acid-based influenza virus detection from throat or nasal swab specimens generally involves the extraction of influenza viral nucleic acids along with a huge background of human nucleic acids. Therefore, to investigate whether our sensor platform could be used to detect the target in clinical settings, a 630-nt H5 cDNA fragment was served as a real sample substituent and spiked into 5 μM human total nucleic acids extracted from human bronchial epithelial (BEAS-2B) cells in the sample buffer, in which the same sandwich hybridization experiments as indicated in Figure 6(a) were performed again and the result is shown in Figure 6(b). Apparently, the similar FRET responses to different target concentrations shown in Figure 6(a) were observed again. This indicates that the detection of H5 target in a non-target complex background is feasible. Notably, the average FRET values (inset of Figure 6(b)) for the target concentrations at 0 nM, 0.5 nM, 5 nM, and 50 nM were decreased to 0.06 (RSD = 12.0%), 0.12 (RSD = 11.3%), 0.19 (RSD = 5.6%), and 0.35 (RSD = 5.9%), respectively. The estimated LOD in this case is about 0.5 nM. The decrease in the LOD is likely due to the decreased hybridization efficiency of the target-probe hybrids in highly non-target rich mixture.

## 4. Conclusions

The sandwich hybridization assay with a QD-induced FRET reporter system has been successfully integrated with a homemade optical sensor composed of an UV LED, a miniature CCD spectrophotometer and a disposable ITO glass slide. With the prototype sensor, label-free H5 target sequences (60-mer DNA or 630-nt cDNA) have been successfully detected. The target concentrations ranging from 0.5 nM to 1 μM has been correlated with both the QD emission decrease at 653 nm and dye emission increase at 690 nm. In the assays, higher dye-to-QD ratios result in higher FRET efficiencies and FRET values, leading to larger extents of FRET emission shift for target DNA quantification. In addition, since single UV LED light source can simultaneously excite different sizes of QDs to induce different QD-dye FRET signals, the current detection platform can also use more than one QD-dye FRET pair for multiplexed nucleic acid diagnosis. Moreover, with the use of temperature-adjustable ITO glass slide, we can incorporate the function of on-slide isothermal nucleic acid amplification into the sandwich hybridization assay for further enhancing the detection sensitivity. In summary, a new QD-induced FRET sensor platform well-suited for both nucleic acid analysis and on-site zoonosis surveillance is accomplished.
